# A giant paratesticular leiomyoma presenting as a painless scrotal mass: a case report

**DOI:** 10.1097/RC9.0000000000000510

**Published:** 2026-06-03

**Authors:** Noor Al-Buloushi, Yousef Ali, Mohammed Dashty, Maram Wael Hasuneya

**Affiliations:** a Kuwait Urology Board, Department of Urology, Jaber Al-Ahmed Hospital, Kuwait City, Kuwait; b Department of Urology, Mubarak Al-Kabeer Hospital, Kuwait City, Kuwait; c Department, Department of Urology, Mubarak Al-Kabeer Hospital, Kuwait City, Kuwait; d Department of Histopathology, Mubarak Al-Kabeer Hospital, Kuwait City, Kuwait

**Keywords:** case report, leiomyoma, paratesticular tumors, radical orchiectomy, testicular neoplasms, testis-sparing approach

## Abstract

**Introduction and importance:**

Leiomyomas are benign smooth muscle tumors that are rarely found in the genitourinary tract. Paratesticular leiomyomas are exceedingly uncommon, representing a small subset of intrascrotal neoplasms. Intrascrotal tumors comprise a heterogeneous group of lesions, with paratesticular tumors accounting for only about 5% of all testicular masses. Awareness of this rare entity is essential, as it may clinically mimic malignant testicular tumors, potentially leading to unnecessary radical orchiectomy.

**Case presentation:**

We present a case of a 55-year-old male who developed a progressively enlarging intrascrotal mass, ultimately diagnosed as a paratesticular leiomyoma. The lesion arose from soft tissue adjacent to the testis and presented as a firm, painless scrotal swelling. Imaging revealed a well-circumscribed, homogeneous lesion without invasion into the testis. Surgical excision was performed, and histopathological analysis confirmed the diagnosis of leiomyoma, showing interlacing bundles of smooth muscle cells without atypia or mitotic activity.

**Clinical discussion:**

Paratesticular leiomyomas originate from the smooth muscle elements of the spermatic cord, epididymis, tunica albuginea, or surrounding tissues. They are benign, slow-growing lesions that may be clinically indistinguishable from malignant testicular tumors. Imaging modalities, including ultrasound and MRI, are useful for preoperative assessment but cannot reliably differentiate benign from malignant lesions. Definitive diagnosis requires histopathological examination demonstrating typical spindle-shaped cells, minimal atypia, and the absence of necrosis or mitotic figures. Complete local excision provides both diagnostic confirmation and curative management.

**Conclusion:**

Although rare, paratesticular leiomyoma should be considered in the differential diagnosis of paratesticular and intrascrotal masses. Accurate diagnosis, based on histopathological examination, prevents overtreatment. Surgical excision remains the treatment of choice and is associated with an excellent prognosis and minimal recurrence risk.

## Introduction

A variety of masses can arise within the testes and paratesticular structures. These lesions may originate from the scrotum, epididymis, spermatic cord, or its coverings. Paratesticular tumors encompass a broad spectrum of histopathological types, including lipomas, adenomatoid tumors, leiomyomas, fibromas, hemangiomas, neurofibromas, cystadenomas, and malignant tumors[1]. Approximately 70% of paratesticular tumors are benign, with lipomas being the most common, whereas 3–30% are malignant[2]. Overall, paratesticular tumors represent only about one-tenth of all testicular and paratesticular neoplasms[3].

HIGHLIGHTS

- Paratesticular tumors represent a rare subset of scrotal neoplasms.
- Leiomyomas are uncommon benign tumors that can arise in the paratesticular region.
- Owing to their clinical similarity to testicular malignancies, surgical exploration remains the cornerstone for definitive diagnosis and management.

Leiomyomas are the second most common paratesticular tumors after adenomatoid tumors. It is difficult to accurately determine the percentage of their occurrence since paratesticular leiomyoma (PTL) is rarely reported. Although leiomyomas are more frequently found in the uterus, gastrointestinal tract, and skin, their occurrence in the male genitourinary tract is uncommon^[^1,2^]^. Clinically, they generally present as asymptomatic, palpable, and slow-growing scrotal masses[2]. On physical examination, however, they are often indistinguishable from malignant lesions[2]. Their clinical presentation and imaging characteristics may overlap with testicular or paratesticular malignancies, and imaging alone is usually insufficient to reliably differentiate benign from malignant disease[3].

Tumor markers such as lactate dehydrogenase (LDH), beta-human chorionic gonadotropin (β-hCG), and alpha-fetoprotein (AFP) are typically within normal limits in PTLs[2]. In cases where malignancy is suspected, or when the mass is inseparable from the testis, radical orchiectomy remains the gold standard approach[2]. Nevertheless, there is increasing interest in testis-sparing surgery, particularly when intraoperative findings and imaging suggest benign pathology and when preservation of fertility is a concern[3].

In this report, we present the case of a PTL that was initially suspected to be malignant based on both clinical and radiological evaluations but was ultimately diagnosed as benign through histopathological examination. We highlight the diagnostic challenges, imaging features, surgical considerations, and the role of testis preservation in such rare cases. This case report has been prepared in accordance with the SCARE 2025 criteria[4].

## Case presentation

A 55-year-old male, with a medical history of deep vein thrombosis on long-term anticoagulation and no prior surgical history, presented to our outpatient clinic with a painless left scrotal mass of 2 weeks’ duration. The patient did not recall when he first noticed the lesion; however, over the past 2 weeks, he reported a sensation of heaviness and a dragging feeling. He denied urinary symptoms, previous scrotal trauma, or a history of cryptorchidism. His family history was negative for testicular malignancy or genetic syndromes. He is married and has three children.

On examination, the left hemiscrotum contained a firm, non-tender, non-fluctuant mass without palpable lymphadenopathy.

**Figure 1.**
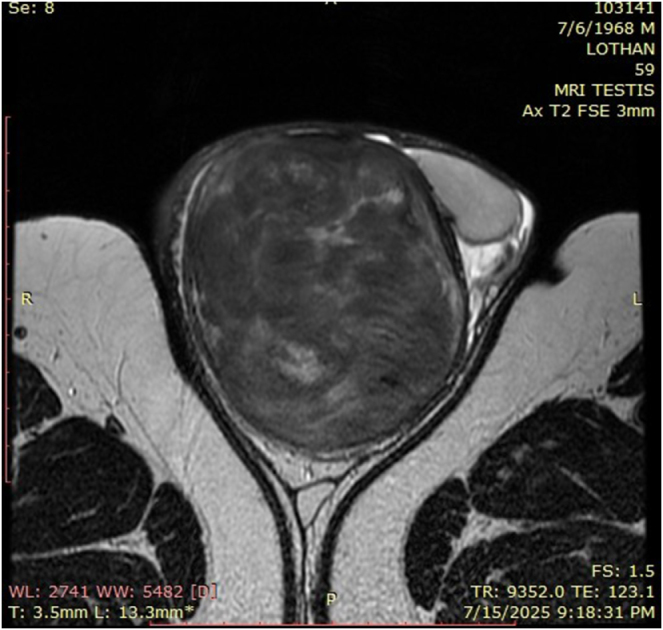
Axial T2-weighted MRI of the scrotum showing a well-circumscribed paratesticular mass adjacent to the right testis. The lesion appears as heterogeneous.

A contrast-enhanced computed tomography (CT) scan was performed, revealing a 9 cm encapsulated, round mass displacing the left testis superiorly, with no internal vascularity. The right testis appeared normal. Further evaluation with magnetic resonance imaging (MRI) demonstrated a large, well-defined, lobulated mass measuring 10.4 cm, compressing the left testis (Fig. 1). The discrepancy in lesion size between the CT and MRI is likely due to the fact that the studies were conducted at two different facilities and interpreted by different radiologists. Hormonal profiles and serum tumor markers, including LDH, β-hCG, and AFP, were all within normal limits. Given the suspicion of malignancy, the patient was counseled regarding surgical management with the possibility of a radical orchiectomy.

**Figure 2.**
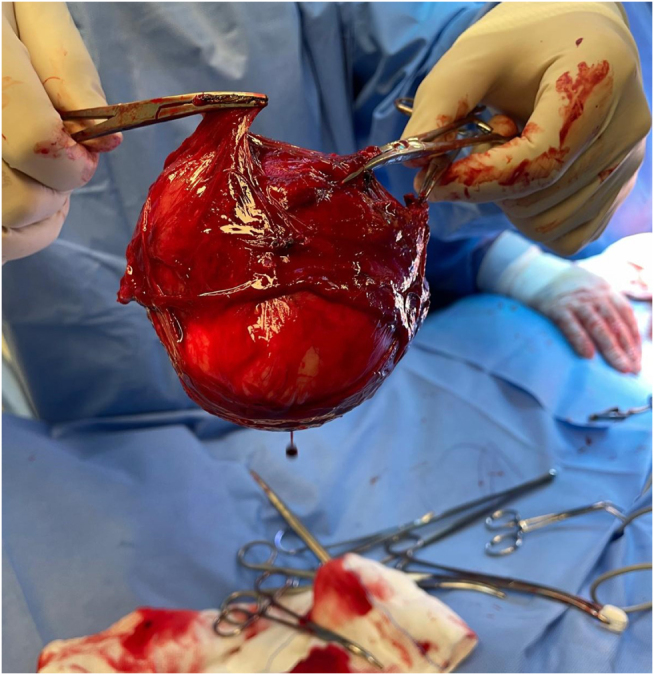
Intraoperative photograph showing the excised paratesticular mass following surgical exploration with preservation of the testis. Final histopathology confirmed the lesion to be a paratesticular leiomyoma.

Intraoperatively, a left inguinal incision extending to a subinguinal length of about 7 cm was performed to enable the delivery of the large mass. After retraction of tissues, the external ring was exposed, and the aponeurosis was incised. The spermatic cord was isolated and clamped with a non-crushing clamp; the vas deferens appeared inseparable from the mass but did not involve the testis. The mass was clamped, ligated, delivered en bloc, and sent to histopathology, while the testis remained preserved (Fig. 2). Given that the mass was well-defined and circumscribed, extratesticular, and lacked any characteristics concerning malignancy, a testis-sparing excision was deemed appropriate. Due to intraoperative bleeding, a drain was placed. Partial closure of the cord was attempted, but complete closure was not feasible owing to the distorted anatomy.

**Figure 3.**
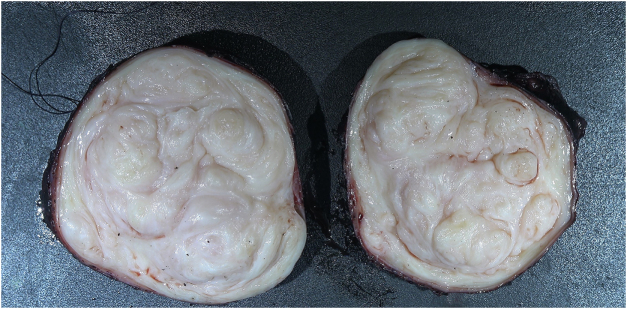
Gross pathological specimen of the excised paratesticular mass after bisection. The cut surface demonstrates a well-circumscribed, firm, white tumor with a characteristic whorled appearance, consistent with a leiomyoma.

Grossly, the specimen consisted of an unoriented, firm, well-circumscribed mass weighing 392.2 g and measuring 11.5 × 11.0 × 5.8 cm. The external surface was intact (inked black), with focal areas of adhesion to the vas deferens (inked green). Serial sectioning revealed a white-tan, whorled cut surface without gross necrosis (Fig. 3).

**Figure 4.**
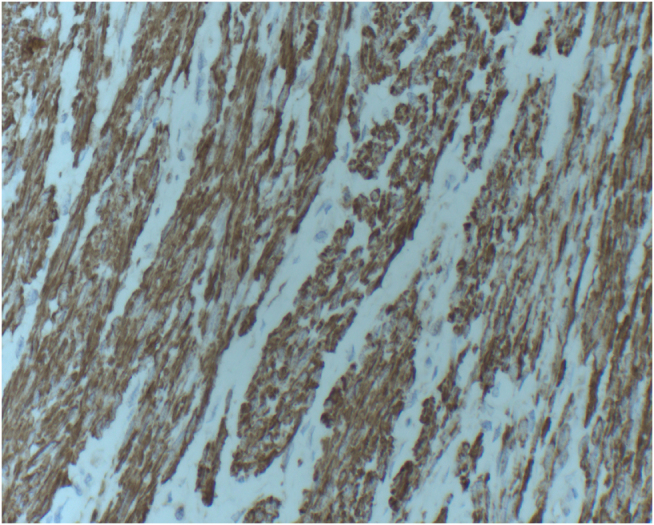
Desmin immunohistochemistry (×20) showing strong cytoplasmic positivity in spindle cells consistent with leiomyoma. Bundles of smooth muscle cells demonstrate.

Microscopically, the tumor was a well-circumscribed smooth muscle neoplasm composed of bland spindle cells arranged in fascicular patterns. The cells demonstrated blunt, cigar-shaped nuclei with eosinophilic cytoplasm, without cytological atypia, mitotic activity, or necrosis. The neoplastic cells showed strong positivity for desmin and smooth muscle actin (SMA), confirming smooth muscle differentiation and the diagnosis of a PTL (Fig. 4). Attached small fragments of benign epididymal and vas deferens tissue were also identified.

The patient had an uncomplicated postoperative course and was later discharged. He presented to the outpatient clinic, asymptomatic, pain-free, and without recurrence of preoperative heaviness. Examination revealed a clean, dry, and well-healed surgical wound with no evidence of infection or hematoma. The testis was preserved with normal position and consistency. Overall, his recovery was smooth, and he continues to do well without any postoperative complications.

## Discussion

Tumors of the paratesticular region may arise from epithelial, mesenchymal, or mesothelial cells[1]. Benign tumors in this location include lipomas, adenomatoid tumors, neurofibromas, and leiomyomas[1]. Among these, leiomyomas are benign mesenchymal encapsulated tumors that can originate from the epididymis, spermatic cord, tunica albuginea, or even the testicular parenchyma[2]. Although exceedingly rare, they are considered the second most common benign paratesticular tumor after adenomatoid tumors[3]. They can be seen at all ages; however, they are most common in the fourth and fifth decades[5].

Clinically, leiomyomas usually present as unilateral, slow-growing, painless scrotal masses, often discovered incidentally during physical examination or imaging^[^2,3^]^.

In contrast to common malignant testicular germ cell tumors, which typically present in young men as intratesticular heterogeneous masses with elevation of tumor markers, PTL usually presents in middle-aged men as a well-circumscribed, extratesticular hypoechoic mass with normal tumor markers[6].

Ultrasound is typically the first-line imaging modality, though findings are often non-specific. It primarily helps distinguish intratesticular from extratesticular lesions but does not reliably differentiate between benign and malignant tumors[3]. Leiomyomas typically appear as solid, hypoechoic, heterogeneous masses[3]. Scrotal MRI may provide additional information, with leiomyomas demonstrating isointense signals on T1-weighted imaging and low signal intensity on T2-weighted imaging[6]. MRI can also delineate extension into surrounding structures, although definitive diagnosis requires surgical excision and histopathological evaluation^[^2,4^]^.

Grossly, leiomyomas are well-circumscribed masses with a firm, white-to-gray capsule[2]. Microscopically, they demonstrate spindle-shaped cells arranged in fascicles, often with areas of hyalinization[2]. Immunohistochemistry further aids in confirmation, with tumor cells showing strong positivity for desmin and SMA and negative staining for CD34 and CD117, excluding other spindle cell neoplasms^[^2,6^]^.

The treatment of choice for PTLs is simple excision or testis-sparing surgery when feasible^[^3,7^]^. However, in cases where the lesion cannot be clearly separated from adjacent structures or when malignancy cannot be excluded, a radical orchiectomy may be warranted^[^3,6^]^. The inguinal approach is preferred to ensure proper oncological control, as preoperative certainty of benignity is rarely absolute[3]. Intraoperative frozen section analysis can be helpful in equivocal cases, potentially avoiding unnecessary radical orchiectomy[6].

The prognosis of PTL is excellent, with very low recurrence rates following complete surgical excision^[^2,3^]^. Table 1 summarizes previously reported cases of testicular leiomyoma, emphasizing variations in presentation, imaging characteristics, and the spectrum of surgical management ranging from testis-sparing excision to radical orchiectomy^[^8–12^]^ .

**Table 1:** Testicular leiomyoma case reports.

Reference	Age	Presentation	Imaging Findings	Treatment
Zouari et al., 2020 (IJSCR)[8]	36 years	Painless testicular mass, several months duration	US: well‑circumscribed, hypoechoic intratesticular lesion	Testis‑sparing excision with frozen section
Haouane et al., 2023 (Cureus)[9]	44 years	Asymptomatic left testicular mass	US: 3.9 cm hypoechoic mass; MRI not done	Radical orchiectomy
Asanad et al., 2020 (Urol Case Rep)[10]	62 years	Incidentally found intratesticular nodule	CEUS: intense homogeneous enhancement; US hypoechoic	Radical orchiectomy
Singh & More, 2022 (JEMDS)[11]	69 years	Slow‑growing mass with recent pain	US: hypoechoic lesion; CT: no metastasis	Bilateral orchidectomy
Seubsang et al., Thai J Surg[12]	43 years	17‑year history of painless swelling	US: well‑defined intratesticular lesion	Orchiectomy

## Conclusion

PTL requires a comprehensive evaluation with clinical examination, imaging, and histopathology to establish a definitive diagnosis. Although clinical findings and imaging may raise suspicion of a malignant neoplasm, it is important to recognize that benign tumors can also present with similar features. Surgical exploration remains the gold standard for diagnosis and treatment.

Whenever feasible, a testis-sparing approach is increasingly advocated, particularly when intraoperative and histological findings support a benign lesion. However, radical orchiectomy may still be warranted when there is a strong suspicion of malignancy, when the tumor cannot be separated from the testis, or when oncological safety cannot be ensured. Prognosis following complete excision is excellent, with very low recurrence rates reported.

## Data Availability

Not applicable.

## Research registration unique identifying number (UIN)

1. Name of the registry: Researchregistry. 2. Unique identifying number or registration ID: researchregistry11528. 3. Hyperlink to your specific registration: https://app.researchregistry.com/research-registry/#home/registrationdetails/68c7327da9920e02a3e278b0/.
